# Long-Term Shrinkage Measurements on Large-Scale Specimens Exposed to Real Environmental Conditions

**DOI:** 10.3390/ma16237305

**Published:** 2023-11-24

**Authors:** Wolfgang Bachofner, Dominik Suza, Harald S. Müller, Johann Kollegger

**Affiliations:** 1 Institute of Structural Engineering, TU Wien, Karlsplatz 13/212-2, 1040 Vienna, Austria; johann.kollegger@tuwien.ac.at; 2 FCP Fritsch, Chiari & Partner ZT GmbH, Marxergasse 1B, 1030 Vienna, Austria; suza@fcp.at; 3 SMP Ingenieure im Bauwesen GmbH, Stephanienstraße 102, 76133 Karlsruhe, Germany; harald.mueller@kit.edu

**Keywords:** concrete, shrinkage, vibrating wire strain gauges, long-term testing, large-scale testing, monitoring

## Abstract

This article presents an experimental testing campaign on large-scale concrete specimens with cross-sectional areas of up to 1 m^2^ and a specimen length of 3 m. The primary goal of the testing campaign was to study the shrinkage behaviour of large-scale specimens exposed to real environmental conditions. Large-scale prismatic concrete specimens were equipped with vibrating wire strain gauges to monitor the strain evolution inside the specimens. To analyse the shrinkage behaviour of the specimens, the thermal strain had to be deducted from the measured strain. To study the influence of seasonal environmental conditions, different specimen production dates (in summer and winter) were examined. The measured shrinkage strains of the large-scale specimens are compared with the results of shrinkage models developed by two engineering entities (*fib* (Fédération Internationale du Béton) and RILEM (International Union of Laboratories and Experts in Construction, Materials, Systems and Structures)). The comparison shows a poor agreement of the measurements with the models, even though the results from the model for small specimens tested in the laboratory under constant environmental condition agree well with the experimental results. This leads to the conclusion that the poor agreement between the measurements and the shrinkage models must be due to the seasonally changing environmental conditions. The comparison of the results from specimens with different production dates shows that different shrinkage behaviour occurs, especially in the first year of measurements.

## 1. Introduction

**Figure 1:**
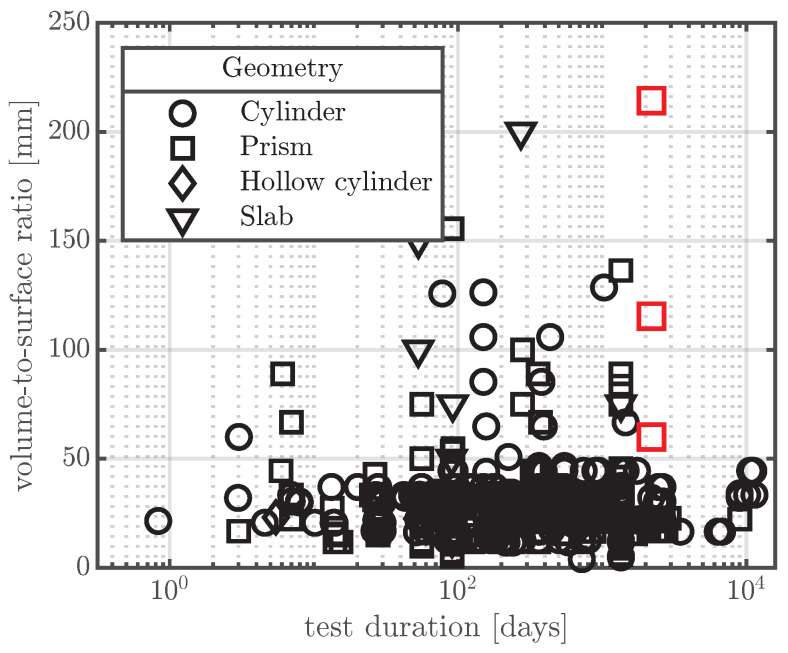
Comparison of the shrinkage test results in the NU database [8] (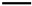) with the large-scale specimens presented in this paper (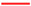) with respect to test duration, specimen size, and specimen shape.

Shrinkage of concrete is usually defined as the load-independent strain due to drying of the material. From an engineering point of view, shrinkage of concrete can be decomposed into drying shrinkage and basic shrinkage [1]. Drying shrinkage is engendered by diffusion of water out of pores, whereas basic shrinkage is observed on sealed specimens when drying is prevented, hence it primarily results from chemical reactions of cement hydration. Therefore, shrinkage is a material property of concrete and has been studied for over a century. Over the past decades, several shrinkage models based on empirical or theoretical considerations have been developed [2,3,4,5] and adapted [6,7] to provide design engineers with guidelines for predicting the shrinkage strain of concrete structures. A huge number of experimental results from shrinkage tests from the past century, carried out by researchers all over the world, is available. Such collections of shrinkage tests are used to either calibrate empirical models or verify theoretical models. The largest available database on creep and shrinkage tests of concrete is the *NU Database of Laboratory Creep and Shrinkage Data* [8]. This database was initially assembled at Northwestern University (NU) in 1978 and expanded to become the NU-RILEM-ACI database in 1992, approved by both RILEM and ACI-209. In 2008, 2015, and 2021, major improvements, restructuring, and verification were carried out [9,10,11]. There are two major issues with the existing shrinkage databases, which were flagged in [12] and more recently mentioned in [13]: (i) The short test duration of the shrinkage tests, and (ii) the small specimen sizes used due to space constraints in laboratory tests. Most of the shrinkage tests contained in the NU database [8] had a duration of less than 1.5 years, and only a very few shrinkage tests had a duration of more than six years. In most of the documented shrinkage tests, the structural member thicknesses do not exceed 0.15 m. In bridges with long spans and in tall buildings, structural members with thicknesses >1 m are often found. The service life of such structures is also generally more than 50 years. It is, however, extremely challenging to extrapolate the measurements in the database to larger member dimensions and longer shrinkage times [14]. To highlight this difficulty, Figure 1 shows the volume-to-surface ratio of the shrinkage tests found in the NU database [8] as a function of the test duration in days. The shrinkage test results presented in this paper are also shown (red squares).

In addition to the fact that the size and service life of concrete structures exceed the size and test duration of the specimens of the shrinkage tests contained in the database, it has also been shown that the influence of seasonal changes in environmental conditions on the results is significant; see, e.g., [15,16]. The influence of seasonal changes in the environmental conditions on the shrinkage behaviour of concrete was studied in [17,18]. Barr et al. [17] analysed the shrinkage behaviour of two bridges with similar cross-sectional dimensions but different production dates. Vandewalle [18] studied the influence of seasonal changes in environmental conditions on concrete cylinders tested in the laboratory. Both testing campaigns [17,18] observed different shrinkage behaviour due to different environmental conditions at the beginning of the measurements, especially in the first month after casting. Therefore, tests on large-scale specimens exposed to real environmental conditions provide the possibility to realistically simulate the behaviour of structural elements in concrete structures and study the influence of seasonal changes in environmental conditions.

To carry out long-term tests on specimens exposed to real environmental conditions, a monitoring concept allowing for continuous measurements is required. Furthermore, to capture the influence of daily temperature cycles, sensors with high resolution and accuracy are needed. Ge et al. [19] tested four types of strain sensors in reinforced concrete beams subjected to thermal loading. The tested sensors included (i) electrical resistance strain gauges (ERSs), (ii) vibrating wire strain gauges (VWSGs), (iii) fibre Bragg gratings (FBGs), and (iv) distributed fibre optic (FO) strain sensors. They concluded that the VWSGs produced the most stable and reliable results among the four tested sensors. The long-term performance of VWSGs has been described by several researchers for different scientific problems; long-term bridge monitoring [15], in situ measurements of the coefficient of thermal expansion of concrete [20,21,22], long-term pre-stress losses in post-tensioned concrete beams [23], and shrinkage measurements [24].

As indicated by Figure 1, there is a need for large-scale and long-term shrinkage testing of concrete. Testing of small-scale specimens at constant environmental conditions in laboratories is of tremendous scientific value for studying the behaviour of concrete and for the calibration and verification of scientific models of concrete shrinkage. To bridge the gap between laboratory tests and real concrete structures, a long-term testing campaign on large-scale specimens (as indicated in Figure 1) was conducted at TU Wien, starting in 2017 [25], using VWSGs as the main measuring devices. To investigate the influence of real environmental conditions, the total shrinkage strain (sum of drying shrinkage and basic shrinkage) and the thermal strain were measured for more than six years. The results of this testing campaign are presented in the following sections.

## 2. Materials and Methods

### 2.1. Experimental Setup

The goal of the long-term testing campaign was to carry out creep and shrinkage tests on large-scale test specimens subjected to real environmental conditions. Initially, four test series were produced, with six large-scale specimens per test series. Every test series consisted of three shrinkage and three creep specimens, resulting in a total number of 24 large-scale specimens. In this publication, the results of the shrinkage specimens of three of the test series (test series S3 is not presented here, since the composition of the concrete was incorrect, which resulted in incorrect slump) are presented.

**Figure 2:**
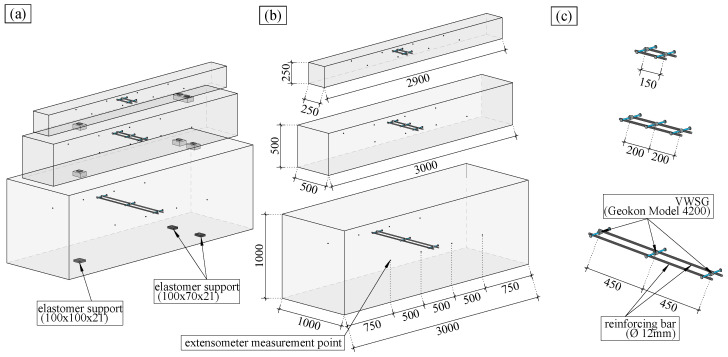
Visualisation of the specimens with dimensions in mm. (**a**) Storage of the specimens. (**b**) Dimensions of the specimens. (**c**) Detailed visualisation of the VWSGs.

The large-scale specimens were designed to cover a broad range of notional member sizes $h_{0}$. According to the *fib* model code 2010 [26], $h_{0}$ is defined as $\frac{2A_{c}}{u}$, where $A_{c}$ is the concrete cross-section and *u* is the perimeter subjected to drying. The test specimens were prisms with a length of 3000 mm for the large and medium specimens and 2900 mm for the small specimens; see Figure 2. The cross-sections are square with edge lengths of 1000 mm for the large specimens, 500 mm for the medium specimens, and 250 mm for the small specimens; see Figure 2.

The first two test series, S1 and S2, were produced in July 2017 and series S4 was produced in February 2018. Series S1 and S4 have the same concrete composition which yields concrete strength class C30/37. A different concrete composition was chosen for test series S2, which yields concrete strength class C50/60.

### 2.2. Production and Storage of the Specimens

**Figure 3:**
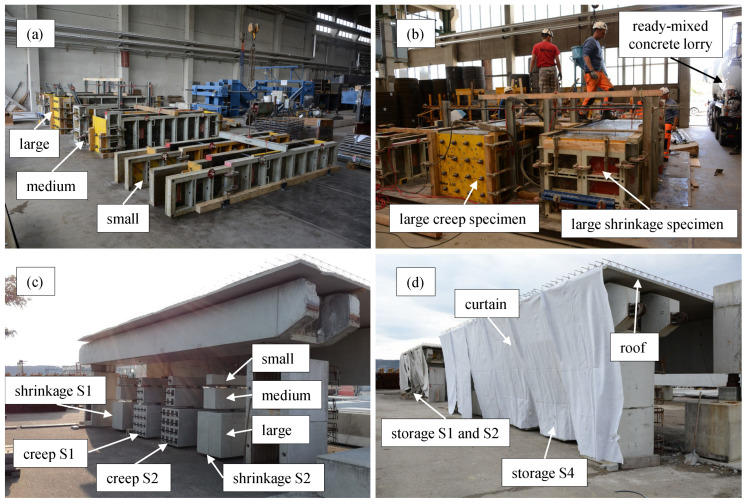
Production and storage of the large-scale specimens. (**a**) Formwork for the specimens of series S1. (**b**) Casting of the large specimens of series S1. (**c**) Storage of the specimens of series S1 and S2 before installation of the curtain. (**d**) Final storage of the test series.

The large-scale specimens were produced to simulate the behaviour of real concrete structures. Therefore, all the production steps such as installing the formwork, casting the concrete, vibrating, curing, and formwork removal was carried out by a construction company; see Figure 3a,b. The production process was the same for all the series. The concrete was cast inside the hall of a precast concrete plant and the stripping was executed on the fourth day after casting. Up to the stripping of the specimens, the tops of the specimens were covered by a protective sheet. The large-scale specimens were stored in the hall of the precast plant until they were transported to their final storage location; see Table 1.

**Figure 4:**
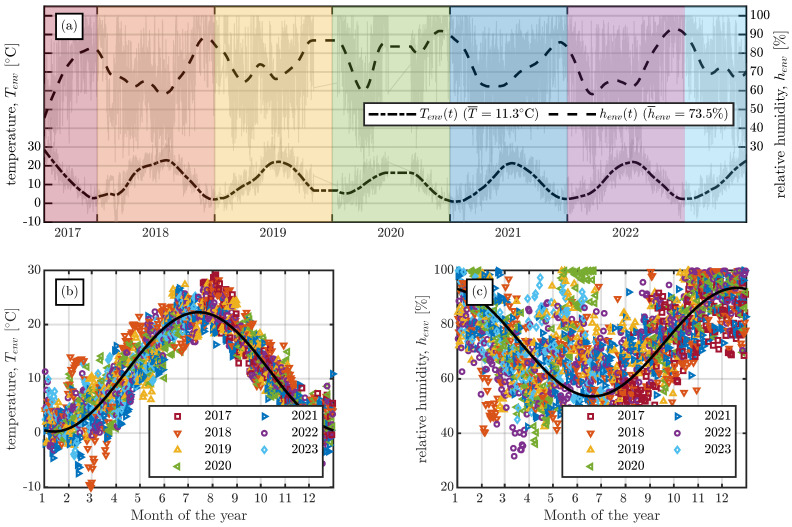
Monitoring of the environmental conditions at the storage yard. (**a**) Temperature $T_{env}\left(t\right)$ and relative humidity $h_{env}\left(t\right)$ data of the whole measurement period. (**b**) Mean daily temperatures recorded at the storage yard compared with a periodic approximation of the temperature. (**c**) Mean daily relative humidity recorded at the storage yard compared with a periodic approximation of the relative humidity.

The final storage location was the storage yard of a precast plant where the specimens were stored outdoors; see Figure 3c. To reduce the influence of direct sunlight and rain, a roof and curtain, as shown in Figure 3d, were installed. The ambient humidity $h_{env}\left(t\right)$ and temperature $T_{env}\left(t\right)$ at the storage yard were monitored continuously. The measurements of the ambient environmental conditions are shown in Figure 4a. To visualise the long-term trend of the measured data, the data were smoothed with a zero-phase filter and a window size of five days (see the dashed-dotted and dashed lines in Figure 4a for $T_{env}\left(t\right)$ and $h_{env}\left(t\right)$, respectively). Figure 4b,c show the mean daily temperatures and relative humidity for each month of the entire measurement period. The measured temperature and humidity values in Figure 4b,c are compared with a harmonic prediction function which oscillates around the mean temperature and relative humidity. The temperature in Figure 4b is described well by the harmonic function, whereas for the relative humidity in Figure 4c the harmonic function gives only a rough estimate. The harmonic function underestimates the relative humidity, especially in the months from May to July.

### 2.3. Concrete Properties

The concrete compositions were chosen to represent two concrete mixes commonly used in Austria. All the series were prepared from a single batch of ready-mixed concrete with compositions as specified in Table 2. Series S1 and series S4 were cast using composition I and series S2 was cast using composition II.

The basic mechanical properties at the age of 28 days are summarised in Table 3. The Young’s modulus *E*, the compressive strength $f_{c}$, and the density ρ were tested on cylinders with Ø150 mm and 300 mm heightaccording to EN-12390-13 [27], EN-12390-3 [28], and EN-12390-7 [29].

The aggregate was the same for both concrete compositions; see Table 2. The lithological character of the coarse aggregate was analysed: the main petrology constituents were vein quartz (54% vol.), limestone (26% vol.), dolomite (9% vol.), and gneiss (6% vol.).

### 2.4. Vibrating Wire Strain Gauges (VWSGs)

To monitor the continuous evolution of the strain inside the large-scale specimens, vibrating wire strain gauges (VWSGs, Geokon Model 4200 [30]) were used. Since the number of VWSGs per series was limited to eight, the large and medium specimens contained three VWSGs and the small specimens contained two VWSGs. As shown in Figure 2, all the large-scale specimens contained two VWSGs which were embedded in the specimen at 50 mm from the concrete surface. The large and medium specimens contained one additional VWSG at the centre of the specimens.

**Figure 5:**
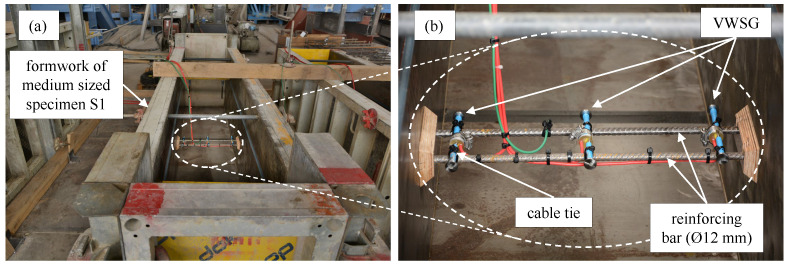
Installation of the VWSGs. (**a**) Formwork of the medium specimen of series S1. (**b**) Detailed picture of the installation of the VWSGs.

The VWSGs had an effective gauge length of 153 mm and a resolution of $1\times 10^{-6}$ m/m with an accuracy of ±0.5% F.S. according to [30]. The sensors were tied to two reinforcing bars with a cable tie, as shown in Figure 5.

The measurements of the VWSGs were recorded using a Campbell Scientific CR6 measurement and control data logger. Every large-scale specimen also had four extensometer measurement points on each side. The extensometer measurements were used to verify the VWSG measurements.

## 3. Theory/Analysis

### 3.1. Determination of Time Zero and Temperature Compensation of the VWSGs

Since concrete is a mixture of cement, aggregate, and water, the hardening of concrete is characterised by the transformation of the liquid phase to a hardened phase, which occurs due to the chemical reactions of the hydration process. The liquid phase of fresh concrete is represented by the cement paste. The hardening of the cement paste can be divided into three stages. The first stage is the dormant period which starts after water has been added to the cement. During the dormant period the cement paste is liquid. The setting period starts when the hydration products start to intersect with each other. During the setting period the cement paste starts to gain stiffness, and at the end of setting the cement paste solidifies. After the setting period finishes, the hardening period starts, during which the cement paste continues to harden and its mechanical strength increases.

**Figure 6:**
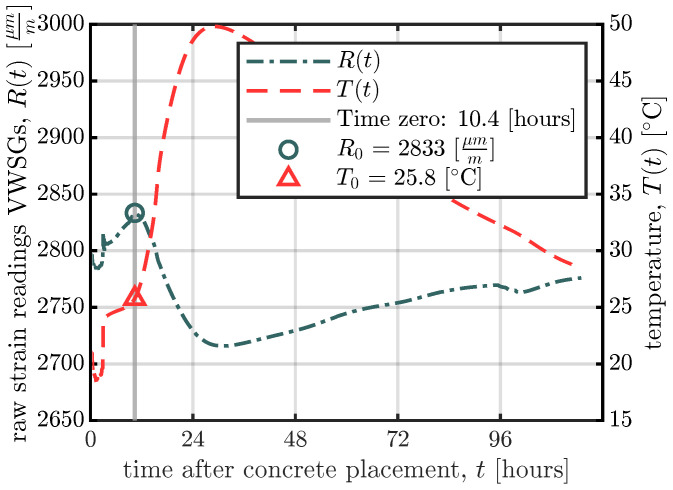
Determination of time zero of the VWSGs according to [31].

The time of setting is therefore important as the point in time at which the VWSGs and the surrounding concrete start to act compositely. Nam et al. [31] defined a procedure to define this point in time, which will hereafter be denoted as time zero. The procedure is based on the following two facts: (i) the raw strains measured by the VWSGs follow the temperature changes while the concrete is plastic and does not exert forces on the VWSGs; and (ii) once the concrete starts setting and the concrete and VWSGs start behaving as a composite, the raw strain readings of the VWSGs and the temperatures move in opposite directions. In the present study, the procedure presented in [31] was used to determine the point in time after which the VWSGs and concrete start to act compositely. Figure 6 shows the determination of time zero and therefore the evolution of the raw strains and temperatures recorded by the VWSG, R(t) and T(t), for the middle VWSG of the large specimen of series S1. Until time zero, the raw strain readings R(t) increase due to the increase in the temperature T(t). At time zero, the raw strain readings R(t) start to decrease although the temperature T(t) still increases, as indicated in Figure 6. The decrease in raw strain readings R(t) at time zero is explained by the fact that from this point in time the VWSG cannot freely expand since the concrete has already started setting and they start to act compositely.

Since the strain readings obtained from the VWSGs are calculated from the changes in the natural frequency of the wire (see, e.g., [32]) and the frequency changes are a result of (i) the strain which the concrete experiences and (ii) the elongation or contraction of the wire owing to changes in temperature, the strain readings have to be adjusted by this temperature-induced elongation/contraction of the wire. This temperature compensation is carried out using Equation (1) proposed by [30]:

(1) $$\varepsilon \left(t\right)=\left(R\left(t\right)-R_{0}\right)\;\cdot \;B+\left(T\left(t\right)-T_{0}\right)\;\cdot \;\alpha_{s}$$

where ε(t) represents the measured strain (μm/m) which the concrete experiences, R(t) and T(t) are the raw strain readings of the VWSG and the temperature measured at time *t*, respectively, $R_{0}$ and $T_{0}$ are the initial reading and the initial temperature at time zero (the point at which the VWSG and the concrete start acting compositely; see Figure 6), respectively, *B* is the batch calibration factor, and $\alpha_{s}$ is the coefficient of thermal expansion of the wire. *B* and $\alpha_{s}$ are 0.98 (-) and 12.2 (μm/m/K), as indicated by the manufacturer.

### 3.2. Separation of the Thermal Strain

**Figure 7:**
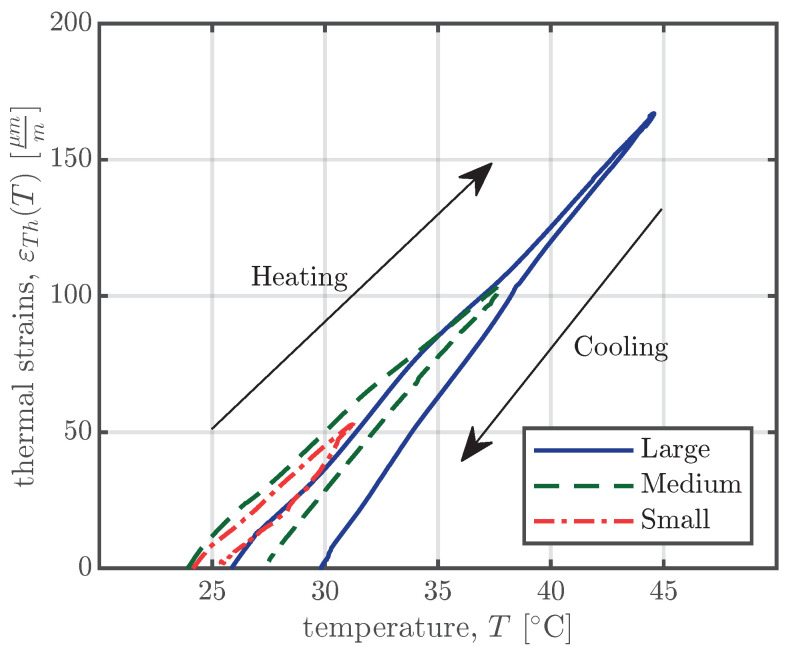
Thermal strain $\varepsilon_{Th}$ as a function of concrete temperature *T* due to hydration. The graph shows the data from the right-hand VWSGs of all specimens of series S1 for the first four days up until the removal of the formwork.

To separate the thermal strain from the measured strain, two different approaches are used to identify the linear coefficient of thermal expansion (CTE). The first approach is used to determine the CTE of the concrete up until the formwork removal. The second approach is used to calculate the CTE during the whole measurement period. For the first approach, the strain measurements of the first four days were used. To determine the CTE at the beginning of the measurements, the hydration heat, as shown in Figure 6, is used. Since both concrete compositions from Table 2 have a sufficiently high water–binder ratio (0.48 and 0.37 for compositions I and II, respectively), the basic shrinkage of the first four days is assumed to be negligibly small. Therefore, the measured strains are assumed to be only the result of heating/cooling due to hydration. Since the slope of this heating/cooling cycle, as shown in Figure 7, represents the CTE, the CTE can be calculated as the secant value. The calculation was carried out for every VWSG for heating and cooling and the mean was determined for every specimen. Table 4 shows a summary of the results for all specimens.

To determine the CTE for the entire measurement period, the following approach was used. The daily mean strains ${\bar{\varepsilon }}_{i}$ and the daily mean temperatures ${\bar{T}}_{i}$ for the *i*-th day of the whole measurement period are calculated. For every daily mean temperature ${\bar{T}}_{i}$, the difference to the daily mean temperature three days before ${\bar{T}}_{i-3}$ is determined. If this difference is greater or equal to ±5 centigrade, the CTE $\alpha_{c,i}$ is calculated with Equation (2):

(2) $$\alpha_{c,i}=\frac{{\bar{\varepsilon }}_{i}-{\bar{\varepsilon }}_{i-3}}{{\bar{T}}_{i}-{\bar{T}}_{i-3}}$$

**Figure 8:**
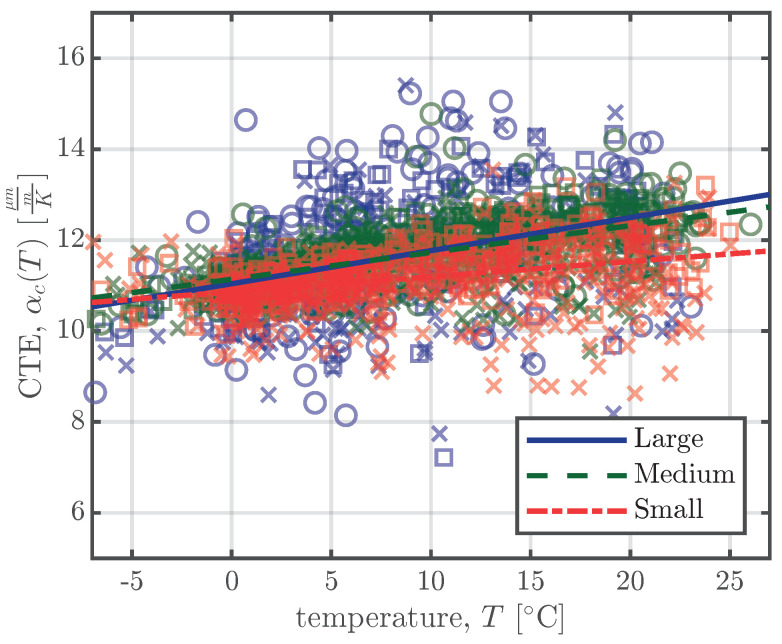
Calculated CTEs, $\alpha_{c,i}$, determined using Equation (2) for the left (×), middle (∘), and right (□) VWSGs of all the specimens of series S1. The linear regression models of Equation (3) are represented by the solid, dashed, and dash-dotted lines.

Figure 8 shows the calculated CTEs $\alpha_{c,i}$ for all the specimens of series S1 as a function of the concrete temperature *T*. *T* in Figure 8 represents the temperature inside the specimens and is defined as the mean of the temperature difference from Equation (2), i.e. $T\;=\;\frac{{\bar{T}}_{i}-{\bar{T}}_{i-3}}{2}$. The calculation procedure of Equation (2) was carried out for all series for the whole measurement period. The beginning of the calculation procedure was set to two months after the production of the specimens to ensure that the large-scale specimens had acclimatised to the environmental conditions at the storage yard. From the calculated CTEs, $\alpha_{c,i}\left(T\right)$, determined using Equation (2), linear regression was used to predict the CTEs as a function of the actual temperature T(t):

(3) $$\alpha_{c}\left(T\left(t\right)\right)=\beta_{0}+\beta_{1}T\left(t\right)$$

The coefficients $\beta_{0}$ and $\beta_{1}$ of the linear regression model in Equation (3) are summarised in Table 5 for all test series and the linear regression is shown in Figure 8 for series S1.

## 4. Results

### 4.1. Strain Measurements

In general, the time-dependent behaviour of concrete can be formulated mathematically within the framework of linear ageing viscoelasticity as follows [33]:

(4) $$\varepsilon \left(t\right)=\int_{0}^{t}J\left(t,t^{\prime }\right)d\sigma \left(t^{\prime }\right)+\tilde{\varepsilon }\left(t\right)$$

The first term of the right-hand side of Equation (4) represents the stress-induced strain, where $J(t,t^{\prime })$ represents the compliance function of concrete, with $t^{\prime }$ denoting the time at loading. The second term of the right-hand side of Equation (4) represents the so-called eigenstrain. The eigenstrain $\tilde{\varepsilon }\left(t\right)$ consists of the thermal strain $\varepsilon_{Th}\left(t\right)$ induced by thermal expansion/contraction, the moisture-related shrinkage strain $\varepsilon_{sh}\left(t\right)$, and the additional strain due to cracking $\varepsilon_{cr}\left(t\right)$.

**Figure 9:**
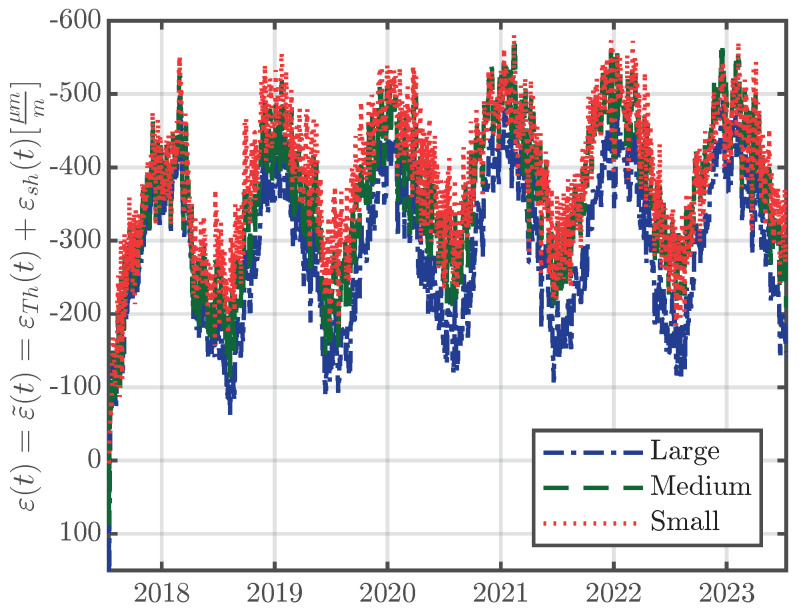
Evolution of the strain ε(t) of the specimens of series S1 over the whole measurement period, according to Equation (1).

Since the large-scale specimens are unloaded and can freely contract or expand, the terms for the stress-induced strain and the strain due to cracking in Equation (4) are assumed to be insignificant and therefore the measured strain ε(t) is effectively just the eigenstrain, $\varepsilon \left(t\right)=\tilde{\varepsilon }\left(t\right)=\varepsilon_{Th}\left(t\right)+\varepsilon_{sh}\left(t\right)$. Figure 9 shows the measured strains ε(t) for the large-scale specimens of series S1 for the whole measurement period.

**Figure 10:**
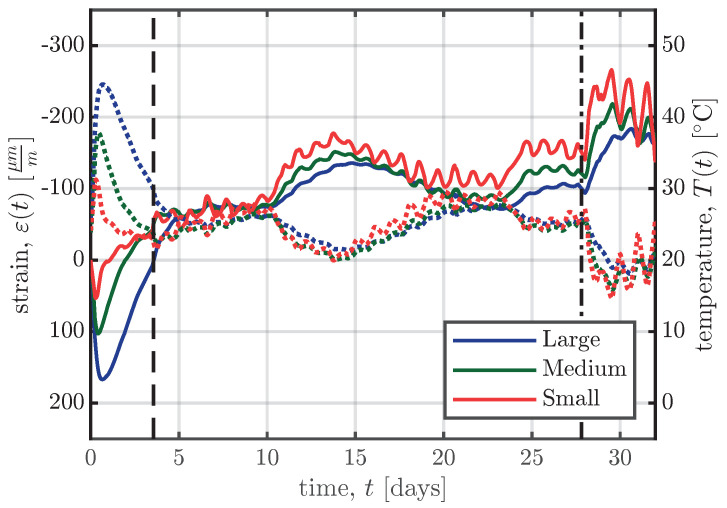
Evolution of strains ε(t) (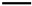) and temperatures T(t) (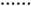) inside the specimens of series S1. The time of formwork removal and the time of transportation to their final storage are represented by the vertical dashed (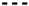) and dash-dotted (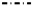) lines, respectively.

Since VWSGs allow measurements to be carried out from the time the sensor and the surrounding concrete start acting compositely, measurements documenting the strain development due to hydration heat can be taken. Figure 10 shows the development of the strain ε(t) and the temperature T(t) inside the specimens for the right-hand sensor of all specimens of series S1 over the first 30 days. The time of the formwork removal and transportation to the final storage are represented as vertical dashed and dash-dotted lines in Figure 10. The time *t* in Figure 10 denotes the time since time zero, as determined in Figure 6.

### 4.2. Shrinkage Strain

**Figure 11:**
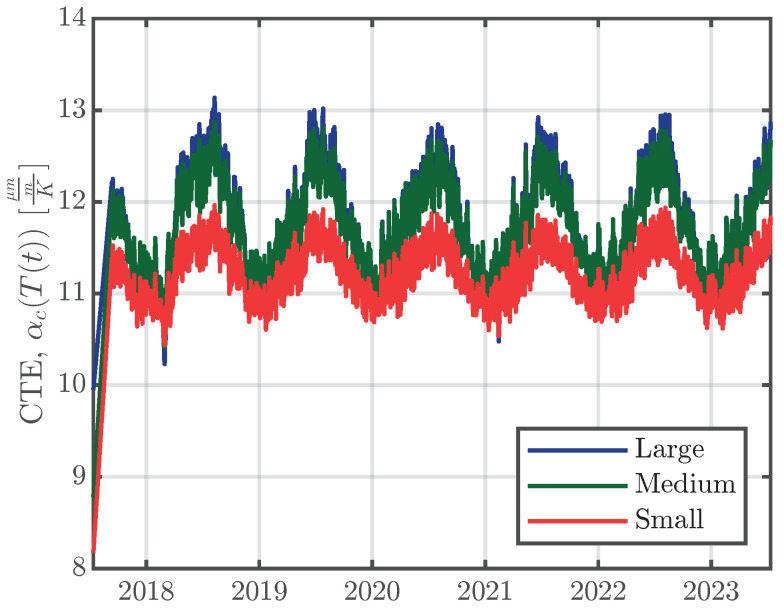
CTEs, $\alpha_{c}\left(T\left(t\right)\right)$, calculated using Equation (3), using the measured temperatures from inside the specimens, T(t), of the right-hand VWSGs of series S1.

For the separation of the thermal strain, $\varepsilon_{Th}$, the early-age CTEs of the specimens (see Table 4), and the CTEs of the whole measurement period (see Table 5) have to be combined. Therefore, the early-age CTEs from Table 4 are taken as the CTEs at the beginning of the measurements (‘time zero’ in Figure 6) and are assumed to increase linearly for the first two months after concrete casting. The CTEs two months after concrete casting are determined using Equation (3), using the temperature T(t) measured inside the specimens. The resulting CTEs for the whole measuring period as a function of the concrete temperatures T(t) inside the specimens are shown in Figure 11 for series S1.

Utilising the calculated CTEs, the separation of the shrinkage strain $\varepsilon_{sh}\left(t\right)$ from the measured strain ε(t) is carried out using Equation (5):

(5) $$\varepsilon_{sh}\left(t\right)=\varepsilon \left(t\right)-\varepsilon_{Th}\left(t\right)=\varepsilon \left(t\right)-\alpha_{c}\left(T\left(t\right)\right)\;\cdot \;\Delta T\left(t\right)$$

**Figure 12:**
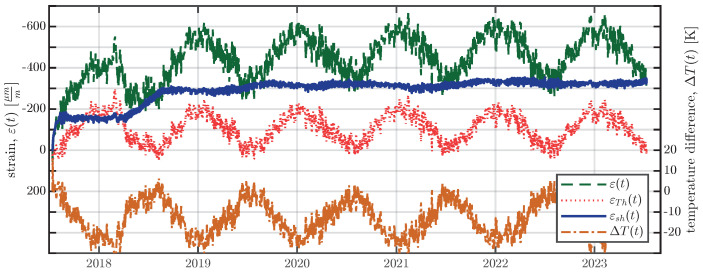
Terms of Equation (5) for the middle VWSGs of the medium specimen of series S1, with ε(t) denoting the measured strain, $\varepsilon_{Th}\left(t\right)$ the thermal strain resulting from the measured temperature T(t) and the CTE, calculated with Equation (3), $\varepsilon_{sh}$ the shrinkage strain obtained from Equation (5), and ΔT the temperature difference used for determining the thermal strain using Equation (5).

Parameter $\alpha_{c}\left(T\left(t\right)\right)$ in Equation (5) denotes the CTE as a function of the current concrete temperature T(t), as shown in Figure 11. ΔT(t) represents the temperature difference between the current temperature T(t) and the initial temperature $T_{0}$. The initial temperature $T_{0}$ is the same as that used for the temperature compensation of the VWSGs in Equation (1) and is shown in Figure 6. From time zero, at which temperature $T_{0}$ is determined, the VWSGs and the concrete undergo elongation/contraction due to temperature changes. The separation of the thermal strain, using Equation (5), is carried out for every VWSG in the large-scale specimens and shown in Figure 12 for the middle VWSG in the medium specimen of series S1.

**Figure 13:**
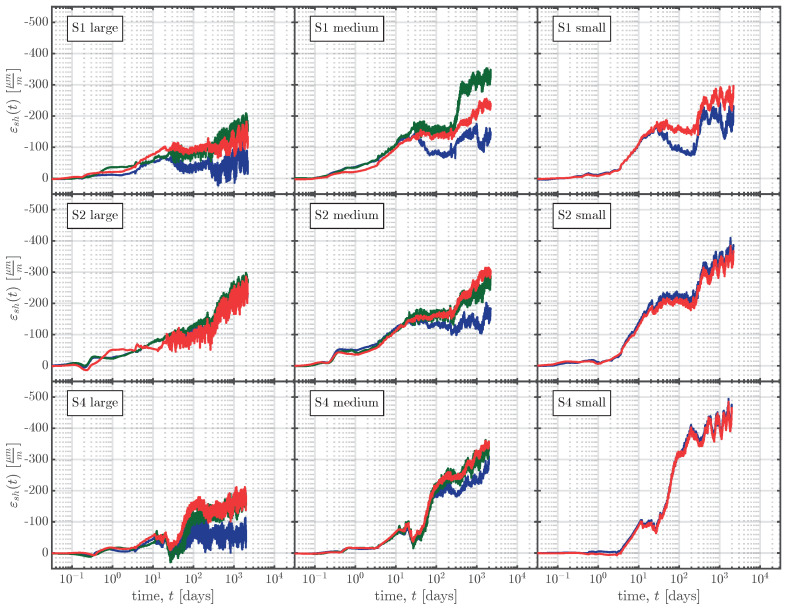
Shrinkage strains $\varepsilon_{sh}\left(t\right)$ determined using Equation (5) for the left-hand (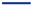), middle (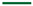), and right-hand (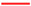) VWSGs of all specimens.

The resulting shrinkage strains for all the VWSGs are summarised in Figure 13. The time in Figure 13 is plotted on a logarithmic scale to visualise the early-age measurements and also capture the long-term trend of the measurements. The time *t* in Figure 13 starts at time zero, as indicated in Figure 6.

## 5. Discussion

### 5.1. Measurements and Calculations

#### 5.1.1. Evaluation of Time Zero of the VWSGs

The procedure presented in Figure 6 is based on the findings of Nam et al. [31] who were the first to use this procedure for VWSGs. Later, Yeon et al. [21] used this procedure to determine the time zero of VWSGs. They also executed a standard penetration resistance test according to [34] to determine the time of setting. In the findings of Yeon et al. [21], the time zero, obtained with the procedure presented in [31], and the final setting times, obtained with the test procedure defined in [34], agreed well with each other for their test specimens. Therefore, Yeon et al. [21] called the time zero of the VWSGs the final setting time.

**Figure 14:**
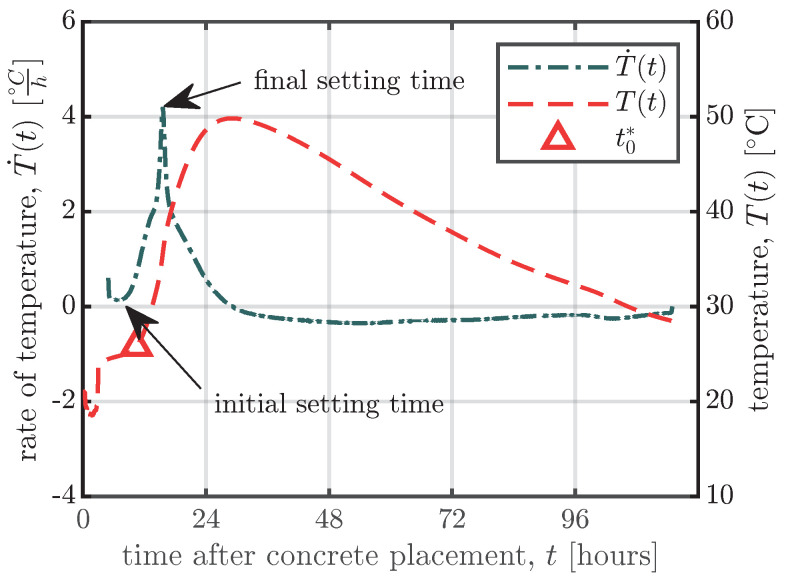
Evolution of temperature T(t) and rate of temperature change $\dot{T}\left(t\right)=\frac{dT(t)}{dt}$ for the first four days after casting. The data shown is for the middle VWSGs of the large specimens of series S1.

Cusson and Hoogeveen [35] used a different approach to determine the time at which stresses start to develop in concrete. They proposed the temperature evolution during the hydration process to be measured as shown in Figure 14. The change in temperature during the dormant period is close to zero, but seven to eight hours after concrete casting, the rate of temperature change starts to increase linearly as shown in Figure 14. Cusson and Hoogeveen [35] propose that the onset and peak of the increase in the temperature rate represent the initial and final setting times, respectively. As shown in Figure 14, the time zero (denoted as $t_{0}^{*}$ in Figure 14) determined according to Nam et al. [31] is close to the initial setting time indicated by the rate of temperature change according to [35].

If it is important to know the exact values of the initial and final setting times, standardised tests have to be performed; see, e.g., [34,36]. For long-term measurements, it suffices to know at what point the VWSGs and the surrounding concrete begin to act compositely. As indicated in Figure 14, $t_{0}^{*}$ lies somewhere between the initial and final setting times of the concrete.

#### 5.1.2. Coefficient of Thermal Expansion

**Figure 15:**
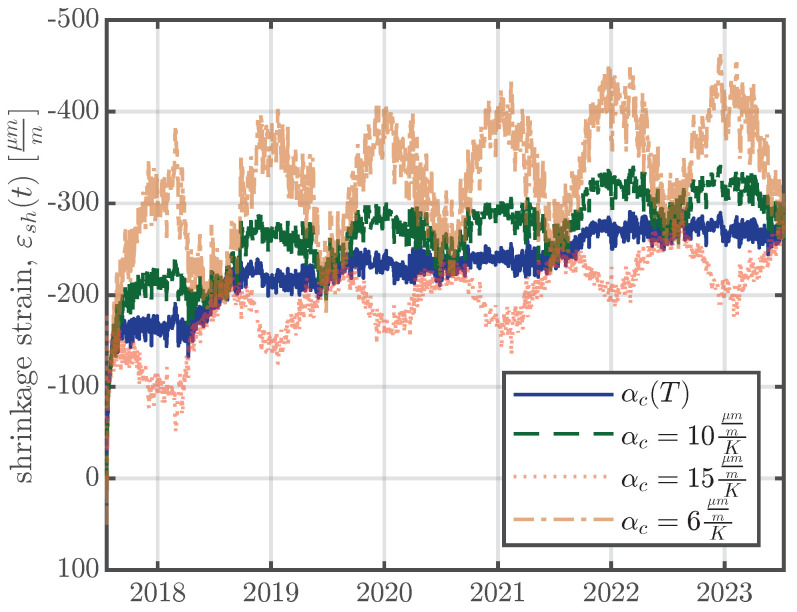
Temperature compensation according to Equation (5) with different CTEs for the medium specimen of series S2 (middle VWSGs).

The CTE of concrete, $\alpha_{c}$, plays a significant role in the process of the separation of the thermal strain. The European standard for concrete structures, Eurocode 2 [6], stipulates a constant value for the CTE of concrete, $\alpha_{c}=10\frac{\frac{\mu m}{m}}{K}$, if no further information is available. *fib* model code 2010 [26] states that the CTE varies between approximately $6\frac{\frac{\mu m}{m}}{K}$ and $15\frac{\frac{\mu m}{m}}{K}$ and recommends a design value of $\alpha_{c}=10\frac{\frac{\mu m}{m}}{K}$. Therefore, the first temperature compensation was carried out using a constant CTE, as recommended in [6,26]. Figure 15 illustrates the temperature compensation according to Equation (5) for different constant CTE values and for a CTE as a function of the concrete temperature, $\alpha_{c}\left(T\right)$, as explained above. The annual shrinkage/swelling cycles visible in Figure 15 were the reason why the CTE for every specimen was determined according to Equation (2). The annual shrinkage/swelling cycles decreased when a temperature-dependent CTE, as defined in Equation (3), was used.

**Figure 16:**
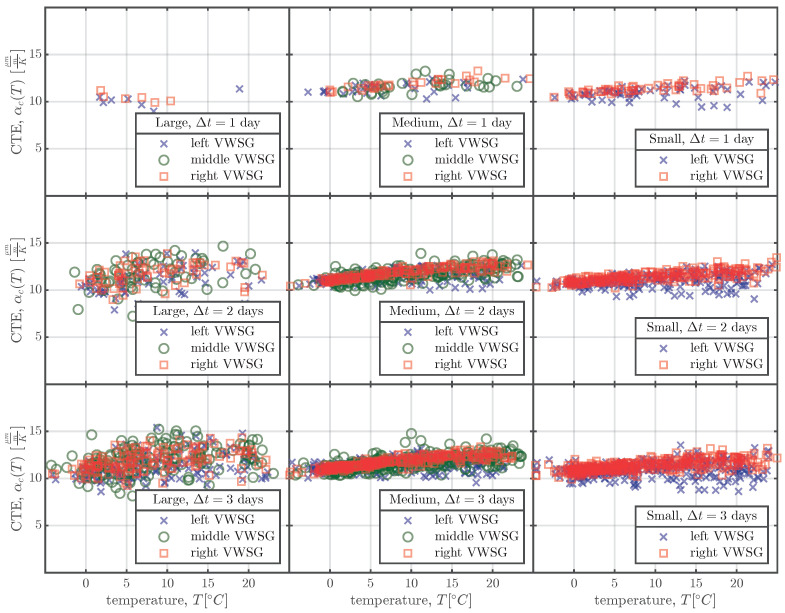
CTEs calculated with Equation (2) for all the specimens of series S1, using different Δt values.

The backward calculation of the CTE for the *i*-th measuring day was executed only if the difference between the daily mean concrete temperature, ${\bar{T}}_{i}$, and the daily mean concrete temperature from three days before, ${\bar{T}}_{i-3}$, was at least ±5 centigrade; see Equation (2). The time difference of Δt=3 days was chosen to ensure that there were enough days for which Equation (2) could be applied, since the scatter in the calculated CTEs is relatively high, as can be seen in Figure 8. For the small specimens, a time difference of Δt=1 days would have sufficed, but because the large specimens are 16 times the mass of the small specimens and therefore need more time to achieve a temperature difference of ±5 centigrade, the larger time difference Δt=3 days was chosen for all the specimens. Figure 16 shows the influence of the time interval Δt (ranging from one to three days) on the calculation approach of Equation (2) for the specimens of series S1. Figure 16 confirms that a time difference Δt of one day would be suitable for the small and medium specimens, but since a larger Δt does not seem to change the value of the calculated CTEs, for simplicity’s sake Δt=3 days was used for all the specimens.

The linear increase in CTE with increasing concrete temperature shown in Figure 8 and Figure 16 can be explained by the dependence of the CTE of cement paste on its internal humidity h(x,t). If we assume that an increase in concrete temperature results in a decrease in internal humidity, the observed behaviour makes sense. As first shown by Meyer [37], and more recently by [38,39], the CTE of cement paste increases with decreasing internal humidity until a value of about h(x,t)≈70% is reached. Since the mean environmental humidity at the storage yard exceeded 70%, as shown in Figure 4, the internal humidity of the large-scale specimens also exceeded 70% and, therefore, a decrease in internal humidity resulted in an increase in the CTE of the cement paste.

**Figure 17:**
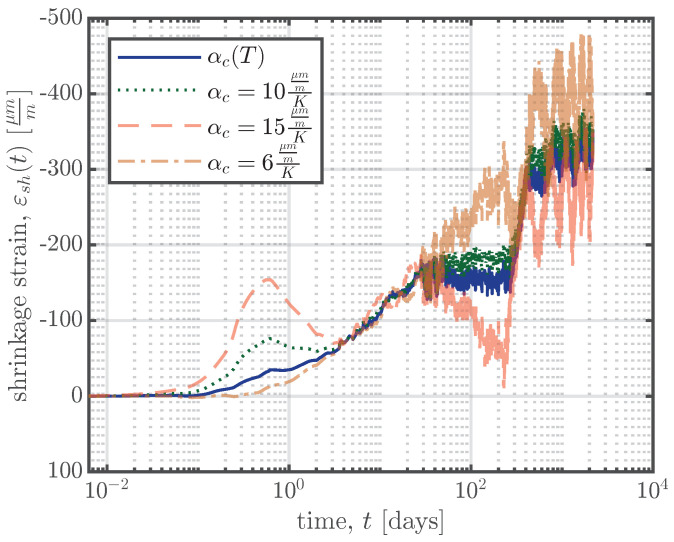
Temperature compensation using Equation (5) for the medium specimens of series S1 (middle VWSGs).

Although a constant value for the CTE of concrete is recommended in various standards [6,26], the CTE at an early concrete age can differ significantly from that of mature concrete, as shown in [40,41]. The early work of Meyer [37] showed the dependence of the CTE on the internal humidity h(x,t) of the concrete. This is particularly relevant for young concrete, where the CTE plays a significant role when the temperature rises or drops due to hydration [42]. Large temperature differences at an early age result in large thermal strain and can cause early-age cracking since the concrete still has not developed its full tensile strength at this stage [43,44]. To measure the CTE in young concrete, several different methods have been used over the past decades; see, e.g., [21,45,46,47]. Since it was not suitable to apply heating/cooling cycles to the large-scale specimens and laboratory measurements for determining the CTE at an early age were not performed, the CTE of the young concrete was determined from the temperature increase and decrease in the material due to hydration, as shown in Figure 7. Figure 17 shows the influence of different CTE values used in the temperature compensation of the medium specimens of series S2 on the shrinkage strain. The time in Figure 17 is plotted on a logarithmic scale to emphasise the influence of the CTE on the temperature compensation in young concrete. Even so, if high-strength concrete mixes with low *w*/*c* ratios are used and therefore the basic shrinkage makes up the largest part of the measured strain, a more sophisticated measurement procedure is needed, as explained in [46], for example.

#### 5.1.3. Extensometer Measurements

**Figure 18:**
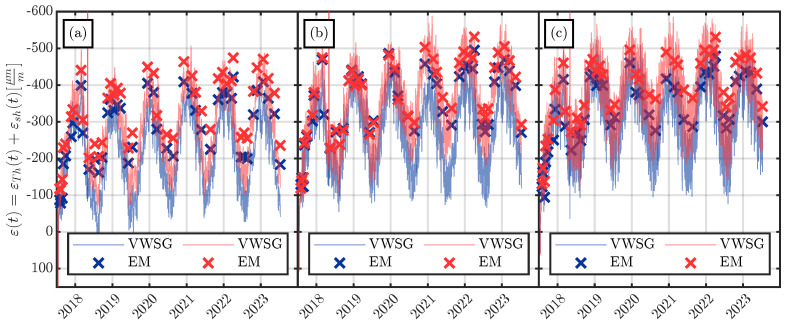
Comparison of the strains ε(t) measured by the VWSGs with the extensometer measurements (EMs) performed on the surfaces of each specimen. The blue measurements (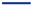) represent the measurements of the VWSG/extensometer on the left side/surface of each specimen and the red measurements (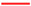) represent the measurements on the right side/surface of each specimen, as shown in Figure 2. (**a**) Large specimens of series S1. (**b**) Medium specimens of series S1. (**c**) Small specimens of series S1.

To verify the measurements of the VWSGs, additional extensometer measurements were carried out over the whole measuring period at relatively regular measurement intervals. Figure 18 shows the comparison of the VWSG and extensometer measurements for the specimens of series S1. As shown in Figure 2, every specimen has four extensometer measurement points, which allows for three extensometer measurements per side face of the specimen. The extensometer measurements (EM) in Figure 18 represent the mean of the three measurements per side face.

As shown in Figure 18, the VWSGs and extensometer measurements agreed well with each other. The difference in the measurements could be due to (i) the different measurement location, since the VWSGs are embedded in the large-scale specimens at a depth of 50 mm while the extensometer measurements are taken at the surface of the specimens; and (ii) the extensometer delivers the mean strain of three measurements with a gauge length of 500 mm per measurement, whereas the VWSGs have an effective measurement length of 153 mm.

Since the extensometer measurements were not taken continuouslyover the whole measuring period, not all of the seasonal variation in the strains was captured by the extensometer. Nevertheless, the extensometer, as a second, independent measurement system, yielded useful information for verifying the VWSG measurements.

### 5.2. Comparison of the Observed Time-Dependent Behaviour with the Predictions of Models from Engineering Societies

The comparison of the measured shrinkage strains with predicted time-dependent deformation values was performed using the concrete shrinkage models of (i) the Fédération Internationale du Béton (*fib*, model code 2010 [26], henceforth called ‘*fib* MC2010’); and (ii) the International Union of Laboratories and Experts in Construction, Materials, Systems and Structures (RILEM [5], henceforth called ’B4s’). Since the *fib* MC2010 shrinkage model [26] is based on the mean concrete compressive strength, the strength-based B4s model [5] and not the full B4 model (which is a composition-based model and therefore requires more input parameters) was chosen. As shown in [12], the B4s model performs almost as well as the B4 model but lacks the capability to predict the influence of the concrete composition under the presence of admixtures.

**Figure 19:**
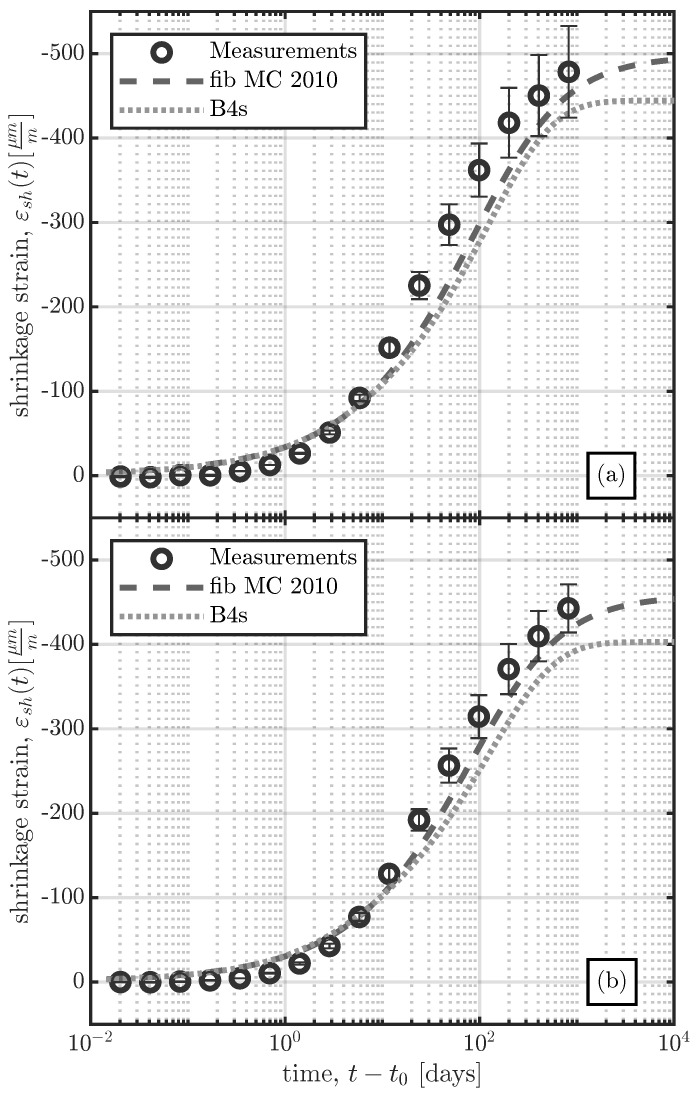
Comparison of shrinkage test results of concrete composition I (CC I) and concrete composition II (CC II) (composition details in Table 2) with the analytical results obtained from *fib* MC 2010 [26] and B4s [5]. The shrinkage tests were performed in a climatic chamber with $T_{env}=20$ °C and $RH_{env}=65\%$. The age at which drying started was $t_{0}=6$ days. (**a**) Concrete composition I. (**b**) Concrete composition II.

Figure 19 shows a comparison of the results of the two models with those of the shrinkage tests carried out in the laboratory. The two concrete compositions specified in Table 2 were tested in a climatic chamber with constant environmental conditions ($T_{env}=20$ °C and $RH_{env}=65\%$) for a duration of over 800 days. The tests were performed on cylinders with a diameter of 150 mm and a height of 300 mm. Every shrinkage test shown in Figure 19 was carried out on two cylinders equipped with three LVDTs (linear variable differential transducers) with a gauge length of 150 mm. Therefore, the measurements displayed in Figure 19 represent the mean of the measurements of six LVDTs. The measurements depicted in Figure 19 represent total shrinkage strain measurements, $\varepsilon_{sh}\left(t\right)$ (sum of the basic shrinkage $\varepsilon_{bs}\left(t\right)$ and the drying shrinkage $\varepsilon_{ds}\left(t\right)$), since the measurements from the large-scale specimens also represent total shrinkage strain measurements. The age at which drying begins was set to $t_{0}=6$ days, since the age of loading in the accompanying creep test was also set to $t^{\prime }=6$ days. In addition to the graphical comparison of the model results with the measurements in Figure 19, a statistical evaluation according to [48] was carried out. The coefficient of variation $\bar{\omega }$ of the deviations of the model results from the test data *j* was calculated using Equation (6):

(6) $${\bar{\omega }}_{j}=\frac{s_{j}}{{\bar{J}}_{j}}=\frac{1}{{\bar{J}}_{j}}{\left[\frac{1}{n-1}\sum_{i=1}^{n}{\left(w_{ij}\Delta_{ij}\right)}^{2}\right]}^{\frac{1}{2}}$$

where

(7) $${\bar{J}}_{j}=\frac{1}{n}\sum_{i=1}^{n}w_{ij}J_{ij}\;\;\mathrm{and}\;\;w_{ij}=\frac{n}{n_{d}n_{1}}$$

where $J_{ij}$ denotes the measured shrinkage strain *i* of dataset number *j*,*n* is the number of data points in dataset *j*, $\Delta_{ij}$ is the deviation of the result of the model from the measured shrinkage strain,$w_{ij}$ are the weights assigned to the data points,$n_{d}$ is the number of decades on the logarithmic time scale spanned by the measurement data in dataset number *j*, and $n_{1}$ is the number of data points in the decade to which point *i* belongs. The coefficients of variation ${\bar{\omega }}_{j}$ for the models and the measurements *j* are summarised in Table 6.

**Figure 20:**
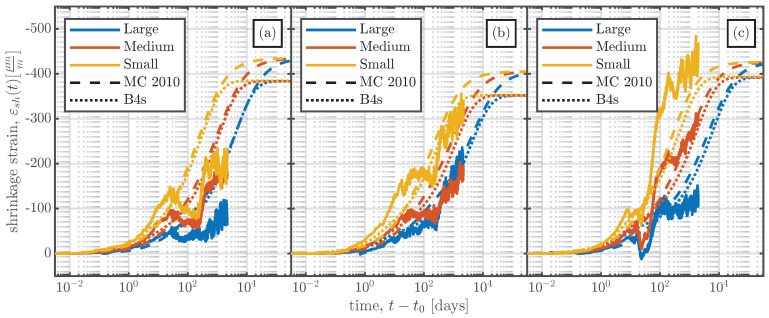
Shrinkage measurements for the large-scale specimens (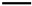) compared with the results of the *fib* MC 2010 [26] (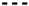) and B4s [5] (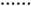) models. The comparison is fulfilled for all the specimens. The specimen size is indicated by different colours; therefore, the large-sized specimens in blue (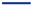), the medium-sized specimens in orange (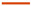), and the small-sized specimens in yellow (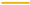). (**a**) Series S1. (**b**) Series S2. (**c**) Series S4.

Figure 20 shows a comparison of the measurements from the large-scale specimens with the shrinkage strain determined using the *fib* MC 2010 and B4s models. Since the models yield the mean shrinkage strain of the cross-section, the mean of the measurements of the VWSGs shown in Figure 13 was calculated and compared with the model results. As both the B4s and the B4 model do not predict basic shrinkage strain to occur before the stripping of the specimen, the comparison was carried out starting at the stripping time $t_{0}$. The statistical evaluation of Equations (6) and (7) for the large-scale specimens was also carried out. The results are summarised in Table 6.

From the comparison of the models with the experimental measurements the following conclusions can be drawn:

- The laboratory measurements, which were performed at a constant temperature and humidity, agree well with the results of both models, with $\bar{\omega }<35\%$, as indicated in Table 6.
- In the large-scale specimens produced in the summer (S1 and S2), the influence of the specimen size on the measured shrinkage strain was relatively small, contrary to the estimates from the considered models.
- The varying environmental conditions over the first 100 days significantly influenced the rate of shrinkage (decreased shrinkage rate for the summer series (S1 and S2) and increased rate for the winter series S4). This is not reflected by either model.
- As can be seen in Table 6, the results from both models yield unsatisfactory results for the large-scale specimens (especially for series S1), due to the reasons mentioned above, i.e. because the effects of seasonal changes in environmental conditions are not captured by the models.

### 5.3. Seasonal Effects and Influence of the Production Date

Although experimental proof of the influence of seasonal changes in environmental humidity and temperature on the shrinkage behaviour of concrete is scarce, some studies have dealt with this topic; see, e.g. [49,50,51,52]. Barr et al. [17] analysed the shrinkage behaviour of bridge segments of two bridges with similar cross-sectional dimensions but different production dates of the monitored segments (late summer and early spring). The bridge segments were manufactured on site and the shrinkage strains of the bridge segments were measured until the segments were installed. The measurement results reported in [17] show significant differences in the evolution of the shrinkage strains over the measurement period up to the installation of the segments (80–140 days) due to the different environmental conditions. The measured shrinkage strains in the bridge segment produced in early spring developed much faster than in the bridge segment produced in late summer. Therefore, the measured shrinkage strain of the bridge segment produced in early spring achieved nearly three times the value of the measured shrinkage strains of the bridge segment produced in the late summer.

A testing campaign to investigate the influence of the production date on the time-dependent behaviour of concrete was performed by Vandewalle [18]. The influence of the date (season) on which the concrete was cast was tested in a climatic chamber. The environmental influence was simulated based on data from weather stations spread all over Belgium. The measurements from these weather stations showed that the seasonal variation in the temperature and relative humidity could be described by means of a sine curve. Therefore, the temperature and humidity in the climate chamber were varied in a sinusoidal manner from a summer period ($T_{env}=20$ °C and $RH_{env}=65\%$) to a winter period ($T_{env}=5$ °C and $RH_{env}=90\%$). The measurements reported in [18] were carried out on cylinders (diameter of 120 mm and height of 300 mm) over more than six years (2300 days). From the shrinkage measurements, Vandewalle [18] observed different shrinkage behaviour in the four specimens cast in different seasons over the first months after casting. In subsequent months, the shrinkage strains measured in the four tests showed the same trend over the measurement period, and after about three years the amount of drying shrinkage strain was nearly the same for all the specimens, irrespective of the season in which they were cast.

**Figure 21:**
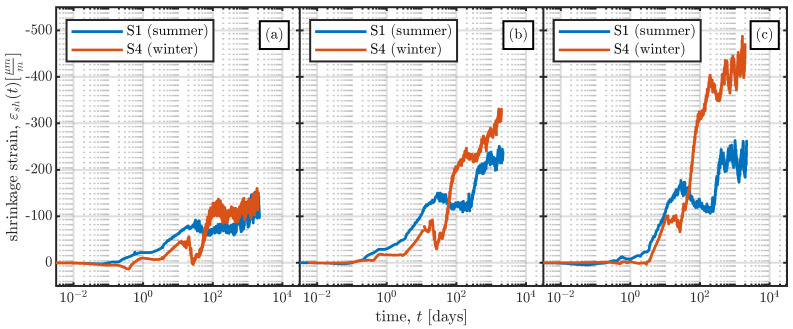
Comparison of the measured shrinkage strains of series S1 (produced in summer 2017) and series S4 (produced in winter 2018). (**a**) Large specimens. (**b**) Medium specimens. (**c**) Small specimens.

Figure 21 shows the measurements of series S1 and S4, which consist of the same concrete mix (see Table 2) and differ in their production dates, as shown in Table 1. The measurements show a different shrinkage behaviour of the specimens during the first year of measurements, as was previously suggested by [17,18]. Contrary to the observations by Vandewalle [18], after more than 2000 days the measured shrinkage strains are not the same for all the specimens. As can be seen in Figure 21, the difference in measured shrinkage strains between the summer and the winter series increases with decreasing specimen size. This increasing difference with decreasing specimen size can be addressed to the influence of variable environmental humidity $h_{env}$. Since shrinkage of concrete represents a diffusion driven process, the influence of variable environmental humidity $h_{env}$ increases with decreasing thickness of the cross-section.

**Figure 22:**
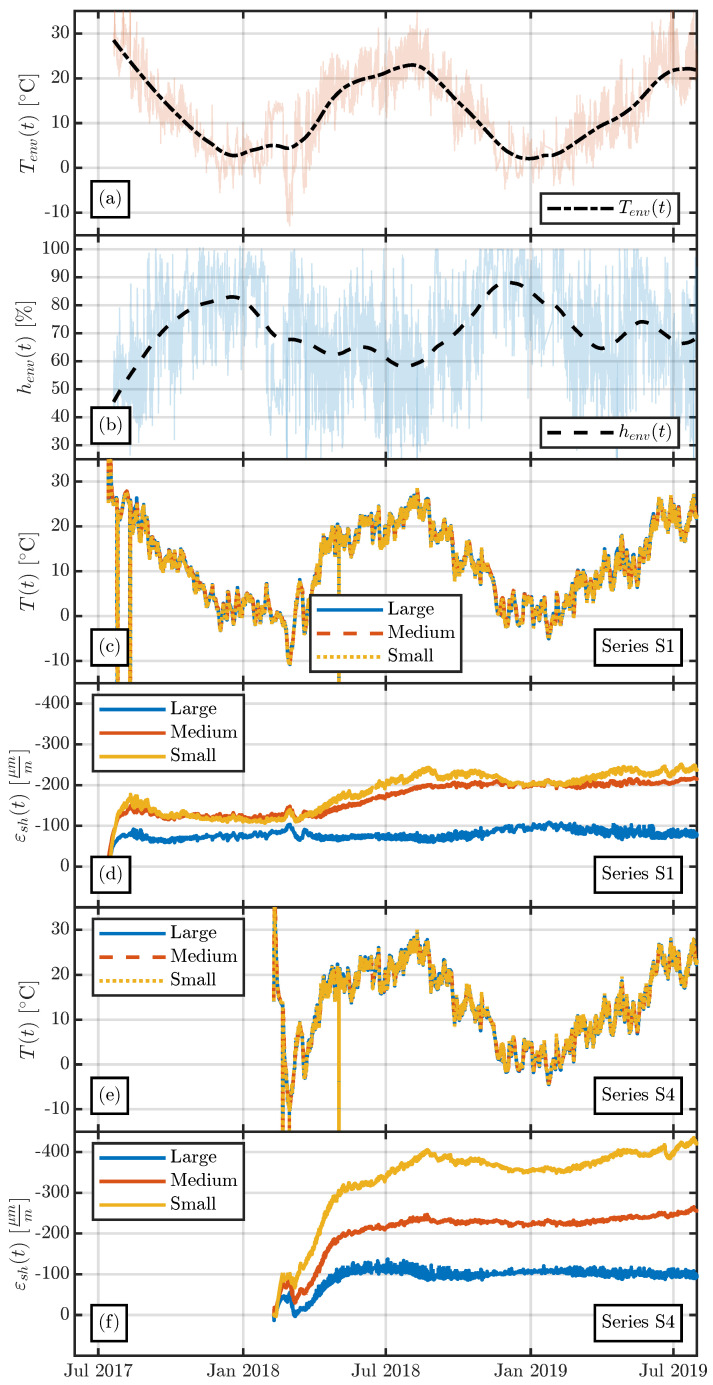
Comparison of the evolution of the shrinkage strains over the first two years of measurements due to the different production dates of series S1 and S4. (**a**) Environmental temperature $T_{env}\left(t\right)$. (**b**) Environmental humidity $h_{env}\left(t\right)$. (**c**) Concrete temperature T(t) of the specimens of series S1. (**d**) Shrinkage strain $\varepsilon_{sh}\left(t\right)$ of the specimens of series S1. (**e**) Concrete temperature T(t) of the specimens of series S4. (**f**) Shrinkage strain $\varepsilon_{sh}\left(t\right)$ of the specimens of series S4.

The difference between the findings of Vandewalle [18] and the measured shrinkage strains in the present study may be due to (i) the yearly variation in environmental humidity in a sinusoidal manner in [18], and (ii) extremely cold temperatures at the end of winter in the first year of measurement (see Figure 4b). As shown in Figure 4c, a sinusoidal approximation function only roughly predicts the monthly variation in the environmental humidity $h_{env}\left(t\right)$ at the storage location of the large-scale specimens. In contrast to the temperature measurements at the storage yard (see Figure 4b), the average daily environmental humidity measurements exhibit large scatter (especially in the months from May to July; see Figure 4c). It can thus be seen that the environmental conditions at the storage yard of the large-scale specimens differ from a perfectly periodic variation in the environmental humidity, as applied in [18]. The possible second reason for the differing shrinkage values taken after more than five years is the extremely low temperature during the first winter of the measurement period. Figure 22 shows the environmental conditions, $T_{env}\left(t\right)$ and $h_{env}\left(t\right)$, the temperature inside the large-scale specimens, T(t), and the shrinkage strain, $\varepsilon_{sh}\left(t\right)$, for series S1 and S4 for the first two years of measurements. The temperature inside the specimens of series S1 decreased nearly linearly for more than six months until reaching a minimum of less than −10 °C in March 2018. This decrease in concrete temperature resulted in a decreasing shrinkage rate, ${\dot{\varepsilon }}_{sh}\left(t\right)\to 0$, until the concrete temperature started to increase again in spring 2018. Series S4 exhibited a different temperature history due to its different production date. After production of the specimens of series S4, the concrete temperature rapidly decreased due to the cold environmental temperatures in February/March 2018 and subsequently increased, as shown in Figure 22. This temperature increase resulted in a significant increase in the shrinkage strain until the concrete temperature started to decrease again at the end of the summer of 2018.

To summarise the findings regarding the influence of different production dates on the shrinkage strain, the following observations can be made:

- The production date has a significant influence on the evolution of the shrinkage strain. Specimens produced in summer exhibited less shrinkage than those produced in winter. Similar observations have previously been documented in [17,18].
- Contrary to the observations of Vandewalle [18], the shrinkage strain of the large-scale specimens did not reach the same shrinkage strain after 2000 days in all specimens, no matter what the production date. The difference between the strains measured in the summer and winter series may be due to the cold winter months during the first year of measurements; see Figure 22.

## 6. Conclusions

Concrete strain measurements were performed on large-scale prismatic specimens over more than six years. The measurements were carried out using vibrating wire strain gauges (VWSGs) embedded in the specimens. The large-scale specimens were unloaded and exposed to real environmental conditions. The analysis of the results of the long-term measurements has led to the following conclusions:

- VWSGs can be used for long-term measurements. In the presented study, VWSGs took strain measurements for over six years and the accuracy of the measurements is confirmed by additional measurements carried out with an extensometer.
- VWSGs allow for early-age concrete strain measurements as soon as the concrete and the sensor start acting compositely (which is some time between the initial and final setting times).
- The coefficient of thermal expansion (CTE) of concrete was back calculated from the measurements of the VWSGs. Two different calculation procedures were used to determine the CTE at an early age and during the whole measurement period.
- The CTE calculated for the whole measurement period shows a dependence on the measured concrete temperature. The calculated CTE values increase with increasing temperature.
- The measured shrinkage strains of the large-scale specimens and the results from analytical models provided by engineering societies (*fib* and RILEM) did not agree well with each other. Since the models do not capture the influence of changing environmental conditions, the measured shrinkage strains of the large-scale specimens were not predicted accurately by the models. Shrinkage strains from the tests carried out in the laboratory agreed well with the results from the models, since the shrinkage tests were performed under constant environmental conditions ($T_{env}=20$ °C and $RH_{env}=65\%$).
- The influence of the production date on the shrinkage strains of the large-scale specimens was investigated. One test series was produced during the summer and a second series with the same concrete mixture was produced in the winter. The measurements show that the evolution of the shrinkage strains significantly differs because of the different production dates. During the first measurement year, the shrinkage strains of the specimens which were produced in winter developed faster and achieved nearly four times the value of the measured shrinkage strains of the specimens which were produced in summer.

## Figures and Tables

**Table 1:** Overview of the testing schedule and the concrete compositions used for the test series.

Series	S1	S2	S4
Production date	13 July 2017	20 July 2017	8 February 2018
Stripping date	17 July 2017	24 July 2017	12 February 2018
Transportation date	10 August 2017	10 August 2017	20 February 2018
Concrete composition	I	II	I

**Table 2:** Concrete composition; quantities in kg/m${}^{3}$.

Concrete Composition	I	II
Cement CEM II A-LL 42.5 N	292	-
Cement CEM II A-LL 42.5 R	-	450
Processed hydraulic additions	73	70
Water	167	185
Aggregate (45% fine, 55% coarse)	1794	1619
Superplasticiser dynamiQ flow L01	2.56	3.9
Air-entraining agent dynamiQ air S-01	0.55	0.78

**Table 3:** Mechanical properties of the concrete at 28 days of age (mean value ± standard deviation).

Property	Unit	S1	S2	S4
Young’s modulus, *E*	GPa	31.6 ± 0.9	32.2 ± 1.1	31.0 ± 0.4
Compressive strength, $f_{c}$	MPa	41.4 ± 0.4	52.9 ± 0.6	44.4 ± 0.1
Density, ρ	kg/dm³	2.30 ± 0.01	2.31 ± 0.02	2.28 ± 0.02

**Table 4:** CTE $\left(\frac{\frac{\mu m}{m}}{K}\right)$ for all the series, determined as secant value from the heating/cooling due to hydration.

Specimen Size	S1	S2	S4
Large	9.69	10.48	10.02
Medium	8.21	10.36	9.14
Small	8.15	10.27	10.59

**Table 5:** Coefficients $\beta_{0}$$\left(\frac{\frac{\mu m}{m}}{K}\right)$ and $\beta_{1}$$\left(\frac{\frac{\mu m}{m}}{{}^{\circ }CK}\right)$ of the linear regression models for the CTEs $\alpha_{c}\left(T\right)$.

		S1	S2	S4
Large	$\beta_{0}$	11.08	11.73	11.83
	$\beta_{1}$	0.07	0.10	0.01
Medium	$\beta_{0}$	11.14	12.05	11.68
	$\beta_{1}$	0.06	0.07	0.06
Small	$\beta_{0}$	10.85	11.58	11.00
	$\beta_{1}$	0.03	0.05	0.03

**Table 6:** Coefficients of variation $\bar{\omega }$ (expressed as percentages). Test data j∈[1,2] denotes the laboratory measurements (see Figure 19) and test data j∈[3,11] denotes the large-scale specimens (see Figure 20).

Test Data *j*		Model	
		**MC 2010**	**B4s**
		$\bar{\omega }$	$\bar{\omega }$
1.	CC I	25.6	33.6
2.	CC II	19.0	31.8
3.	S1 large	271.3	251.6
4.	S1 medium	136.8	123.3
5.	S1 small	163.6	148.8
6.	S2 large	28.5	30.2
7.	S2 medium	125.5	98.0
8.	S2 small	35.9	23.9
9.	S4 large	146.0	113.0
10.	S4 medium	23.1	35.5
11.	S4 small	38.9	49.7

## Data Availability

The data of the shrinkage measurements of the large-scale testing campaign and of the tests carried out in the laboratory are summarised in tabular form in Appendix A. Further data supporting the findings of this study are available on request from the corresponding author.

## Appendix A. Shrinkage Data

**Table A1:** Results of shrinkage tests of concrete composition I and concrete composition II (for composition details see Table 2). The shrinkage tests were performed in a climatic chamber with $T_{env}=20$ °C and $RH_{env}=65\%$ on cylinders with a diameter of 150 mm and a height of 300 mm. The age at the start of drying was $t_{0}=6$ days.

Composition I		Composition II	
$t-t_{0}$	$\varepsilon_{\mathrm{sh}}$	$t-t_{0}$	$\varepsilon_{\mathrm{sh}}$
**(day)**	**(** $\frac{\mu m}{m}$ **)**	**(day)**	**(** $\frac{\mu m}{m}$ **)**
0.02	1.06	0.02	0.06
0.04	1.84	0.04	0.27
0.08	−0.59	0.08	−0.56
0.17	−0.42	0.17	−2.00
0.34	−5.23	0.34	−4.44
0.70	−12.66	0.69	−10.54
1.41	−26.41	1.41	−22.02
2.87	−51.09	2.85	−42.69
5.82	−92.09	5.79	−77.09
11.82	−151.62	11.74	−128.11
23.98	−225.27	23.81	−192.08
48.65	−297.38	48.30	−256.48
98.72	−362.03	97.98	−314.49
200.33	−418.16	198.75	−370.78
406.50	−450.37	403.16	−409.74
824.87	−478.49	817.79	−442.78

**Table A2:** Shrinkage measurements of the large-scale specimens. The measurements are summarised for the large specimens (L), medium specimens (M), and small specimens (S) (dimensions see Figure 2) of all series. The age at the start of drying was $t_{0}=4$ days.

	Series S1				Series S2				Series S4		
	**L**	**M**	**S**		**L**	**M**	**S**		**L**	**M**	**S**
$t-t_{0}$	$\varepsilon_{\mathrm{sh}}$	$\varepsilon_{\mathrm{sh}}$	$\varepsilon_{\mathrm{sh}}$	$t-t_{0}$	$\varepsilon_{\mathrm{sh}}$	$\varepsilon_{\mathrm{sh}}$	$\varepsilon_{\mathrm{sh}}$	$t-t_{0}$	$\varepsilon_{\mathrm{sh}}$	$\varepsilon_{\mathrm{sh}}$	$\varepsilon_{\mathrm{sh}}$
**(day)**	**(** $\frac{\mu m}{m}$ **)**	**(** $\frac{\mu m}{m}$ **)**	**(** $\frac{\mu m}{m}$ **)**	**(day)**	**(** $\frac{\mu m}{m}$ **)**	**(** $\frac{\mu m}{m}$ **)**	**(** $\frac{\mu m}{m}$ **)**	**(day)**	**(** $\frac{\mu m}{m}$ **)**	**(** $\frac{\mu m}{m}$ **)**	**(** $\frac{\mu m}{m}$ **)**
0.06	−1.71	−2.59	−3.43	0.06	−0.31	−0.55	−0.79	0.06	−1.00	−1.20	−0.30
0.08	−1.94	−4.42	−7.82	0.08	−0.51	−0.94	−1.74	0.08	−1.60	−1.46	−2.80
0.11	−2.45	−4.26	−8.43	0.11	−0.71	−1.37	−2.65	0.11	−2.04	−1.44	−2.76
0.15	0.04	−4.61	−9.77	0.15	−1.27	−2.09	−3.66	0.14	−1.14	−2.77	−3.69
0.20	−0.68	−5.56	−10.97	0.20	−3.58	−4.80	−5.56	0.19	0.19	−3.16	−4.51
0.26	−3.79	−7.95	−14.10	0.26	−2.64	−6.65	−6.19	0.25	−0.64	−3.92	−5.50
0.35	−5.82	−8.74	−15.11	0.35	−3.52	−7.80	−14.99	0.34	−1.44	−4.86	−7.37
0.47	−7.00	−9.55	−16.69	0.47	−5.02	−9.37	−18.50	0.45	−2.35	−6.07	−10.27
0.63	−8.22	−10.67	−18.35	0.63	−5.17	−9.61	−22.97	0.60	−5.03	−7.68	−14.07
0.84	−9.35	−13.13	−22.18	0.84	−0.80	−11.49	−27.02	0.80	−6.51	−9.54	−17.90
1.13	−11.05	−16.76	−27.54	1.12	−6.30	−14.71	−33.94	1.07	−8.14	−11.86	−21.72
1.51	−15.27	−18.15	−26.94	1.50	−8.26	−21.32	−41.42	1.43	−11.49	−14.05	−27.58
2.02	−15.55	−23.09	−38.20	2.01	−10.06	−24.31	−47.94	1.91	−13.66	−17.77	−34.64
2.70	−18.76	−27.47	−44.22	2.69	−14.13	−31.59	−58.49	2.56	−16.97	−22.10	−43.00
3.61	−22.55	−33.01	−51.83	3.60	−15.36	−36.01	−64.49	3.41	−21.51	−27.31	−53.79
4.83	−25.15	−40.32	−63.89	4.82	−23.79	−41.72	−76.98	4.56	−25.48	−34.23	−67.34
6.46	−30.03	−50.08	−78.96	6.45	−27.64	−50.11	−94.89	6.09	−30.50	−43.45	−82.84
8.64	−35.83	−64.05	−97.91	8.63	−34.65	−58.78	−111.78	8.38	−34.73	−58.02	−100.20
11.57	−40.31	−70.27	−104.22	11.54	−33.71	−63.80	−127.70	10.85	−30.02	−50.32	−86.07
15.48	−46.10	−75.80	−114.42	15.44	−40.83	−78.32	−152.13	14.49	−29.48	−60.39	−88.29
20.71	−42.80	−83.04	−129.16	20.66	−37.84	−73.45	−131.92	19.34	−18.32	−34.04	−74.28
27.71	−43.37	−87.00	−129.30	27.65	−48.90	−86.31	−158.87	25.83	6.48	−24.28	−91.25
37.07	−41.07	−91.99	−130.56	36.99	−55.10	−95.10	−184.18	34.49	−6.02	−33.70	−113.15
49.60	−30.51	−75.27	−109.35	49.49	−54.72	−90.46	−181.28	46.06	−22.40	−69.08	−161.64
66.37	−33.05	−64.25	−93.47	66.21	−46.89	−77.62	−167.55	61.50	−65.69	−128.76	−240.69
88.81	−29.43	−70.75	−103.35	88.59	−64.87	−89.36	−179.60	82.12	−87.71	−174.24	−299.64
118.83	−40.15	−68.57	−88.32	118.53	−69.63	−84.70	−170.93	109.65	−98.57	−188.51	−317.50
159.00	−36.89	−63.18	−85.82	158.59	−76.96	−89.01	−170.37	146.41	−105.99	−202.09	−353.06
212.74	−51.33	−73.25	−89.66	212.18	−87.10	−90.23	−166.94	195.50	−89.79	−211.16	−389.34
284.66	−35.29	−86.81	−133.86	283.89	−72.12	−94.60	−211.60	261.04	−77.92	−212.04	−377.70
380.88	−35.94	−137.32	−191.31	379.83	−109.89	−119.14	−268.84	348.57	−100.26	−207.42	−353.53
509.63	−54.48	−141.85	−172.29	508.19	−154.28	−131.19	−251.79	465.44	−96.20	−218.70	−388.45
681.91	−47.20	−154.92	−200.18	679.94	−129.73	−123.60	−269.33	621.49	−103.95	−243.43	−397.52
912.41	−89.54	−174.74	−188.47	909.72	−185.47	−144.83	−270.50	829.86	−90.43	−259.70	−438.50
1220.84	−91.71	−165.43	−166.61	1217.17	−195.27	−159.00	−297.28	1108.10	−110.09	−255.96	−375.40
1633.53	−93.04	−178.57	−189.24	1628.51	−206.05	−185.20	−305.44	1479.62	−124.93	−285.69	−421.24
2185.73	−69.07	−183.58	−230.96	2178.86	−194.98	−185.11	−339.78	1975.71	−109.30	−311.60	−466.18

## References

[1] Müller H.S., Anders I., Breiner R., Vogel M. (2013). Concrete: Treatment of types and properties in fib Model Code 2010. Struct. Concr..

[2] Müller H.S., Kvitsel V. (2002). Kriechen und Schwinden von Beton. Beton Stahlbetonbau.

[3] Müller H.S., Acosta F., Kvitsel V. (2021). Modelle zur Vorhersage des Schwindens und Kriechens von Beton—Teil 1: Analyse des Schwindmodells in DIN EN 1992-1-1:2011 und neuer Ansatz im Eurocode 2 prEN 1992-1-1:2020. Beton Stahlbetonbau.

[4] Bažant Z.P., Baweja S. (1995). Creep and Shrinkage Prediction Model for Analysis and Design of Concrete Structures: Model B3. Mater. Constr..

[5] Bažant Z.P., Hubler M.H., Wendner R. (2015). Model B4 for creep, drying shrinkage and autogenous shrinkage of normal and high-strength concretes with multi-decade applicability. Mater. Struct..

[6] CEN (European Committee for Standardization) (2014). Eurocode 2: Design of Concrete Structures—Part 1-1: General Rules and Rules for Buildings.

[7] ACI (American Concrete Institut) (2008). Guide for Modeling and Calculating Shrinkage and Creep in Hardened Concrete.

[8] (2021). NU Database of Laboratory Creep and Shrinkage Data. http://www.civil.northwestern.edu/people/bazant/downloads.html.

[9] Bažant Z.P., Li G.H. (2008). Comprehensive Database on Concrete Creep and Shrinkage. Aci Mater. J..

[10] Hubler M.H., Wendner R., Bažant Z.P. (2015). Comprehensive Database for Concrete Creep and Shrinkage: Analysis and Recommendations for Testing and Recording. Aci Mater. J..

[11] Šmilauer V., Havlásek P., Dohnalová L., Wan-Wendner R., Bažant Z.P. Revamp of Creep and Shrinkage NU Database. Proceedings of the The Biot-Bažant Conference.

[12] Hubler M.H., Wendner R., Bažant Z.P. (2015). Statistical justification of Model B4 for drying and autogenous shrinkage of concrete and comparisons to other models. Mater. Struct..

[13] Bažant Z.P., Nguyen H.T., Dönmez A.A. (2022). Scaling in size, time and risk—The problem of huge extrapolations and remedy by asymptotic matching. J. Mech. Phys. Solids.

[14] Bažant Z.P., Donmez A. Extrapolation of Test Data in Time, Size and Risk: A Challenge for Concrete Design Codes. Proceedings of the IABSE Symposium Prague, 2022: Challenges for Existing and Oncoming Structures.

[15] Kolínský V., Vítek J.L. (2019). Verification of numerical creep and shrinkage models in an arch bridge analysis. Struct. Concr..

[16] Herbers M., Wenner M., Marx S. (2023). A 576 m long creep and shrinkage specimen—Long-term deformation of a semi-integral concrete bridge with a massive solid cross-section. Struct. Concr..

[17] Barr B.I.G., Vitek J.L., Beygi M.A. (1997). Seasonal shrinkage variation in bridge segments. Mater. Struct..

[18] Vandewalle L. (2000). Concrete creep and shrinkage at cyclic ambient conditions. Cem. Concr. Compos..

[19] Ge Y., Elshafie M.Z.E.B., Dirar S., Middleton C.R. (2014). The response of embedded strain sensors in concrete beams subjected to thermal loading. Constr. Build. Mater..

[20] Jeong J.H., Zollinger Dan G., Lim J.S., Park J.Y. (2012). Age and Moisture Effects on Thermal Expansion of Concrete Pavement Slabs. J. Mater. Civ. Eng..

[21] Yeon J.H., Choi S., Won M.C. (2013). In situ measurement of coefficient of thermal expansion in hardening concrete and its effect on thermal stress development. Constr. Build. Mater..

[22] Yeon J.H., Choi S., Won M.C. (2013). Evaluation of zero-stress temperature prediction model for Portland cement concrete pavements. Constr. Build. Mater..

[23] Guo T., Chen Z., Lu S., Yao R. (2018). Monitoring and analysis of long-term prestress losses in post-tensioned concrete beams. Measurement.

[24] Choi S. (2017). Internal relative humidity and drying shrinkage of hardening concrete containing lightweight and normal-weight coarse aggregates: A comparative experimental study and modeling. Constr. Build. Mater..

[25] Suza D. (2020). Einfluß des Maßstabseffekts und der Umgebungsbedingungen auf das Kriechen und Schwinden von Beton. Ph.D. Thesis.

[26] Fédération internationale du béton (fib) (2013). fib Model Code for Concrete Structures 2010.

[27] (2014). CEN (European Committee for Standardization). Testing Hardened Concrete—Part 13: Determination of Secant Modulus of Elasticity in Compression.

[28] (2009). CEN (European Committee for Standardization). Testing Hardened Concrete—Part 3: Compressive Strength of Test Specimens.

[29] (2009). CEN (European Committee for Standardization). Testing Hardened Concrete—Part 7: Density of Hardened Concrete.

[30] (2019). Geokon, Model 4200 Vibrating Vire Strain Gauges Instruction Manual. https://www.geokon.com/4200-Series.

[31] Nam J.H., Kim D.H., Choi S., Won M.C. (2007). Variation of Crack Width over Time in Continuously Reinforced Concrete Pavement. Transp. Res. Rec..

[32] Mang H.A., Hofstetter G. (2018). Festigkeitslehre.

[33] Bažant Z.P., Jirásek M. (2018). Creep and hygrothermal effects in concrete structures. Solid Mechanics and its Applications.

[34] ASTM (American Society for Testing and Materials) (2008). Standard Test Method for Time of Setting of Concrete Mixtures by Penetration Resistance.

[35] Cusson D., Hoogeveen T. (2007). An experimental approach for the analysis of early-age behaviour of high-performance concrete structures under restrained shrinkage. Cem. Concr. Res..

[36] (2008). CEN (European Committee for Standardization). Methods of Testing Cement—Part 3: Determination of Setting Times and Soundness.

[37] Meyer S.L. Thermal Expansion Characteristics of Hardened Cement Paste and of Concrete. Proceedings of the Thirtieth Annual Meeting of the Highway Research Board.

[38] Grasley Z.C., Lange D.A. (2007). Thermal dilation and internal relative humidity of hardened cement paste. Mater. Struct..

[39] Aili A., Maruyama I., Vandamme M. (2023). Thermal Expansion of Cement Paste at Various Relative Humidities after Long-term Drying: Experiments and Modeling. J. Adv. Concr. Technol..

[40] Bjøntegaard Ø., Sellevold E.J. (2001). Interaction between thermal dilation and autogenous deformation in high performance concrete. Mater. Struct..

[41] Sellevold E.J., Bjøntegaard Ø. (2006). Coefficient of thermal expansion of cement paste and concrete: Mechanisms of moisture interaction. Mater. Struct..

[42] Wyrzykowski M., Lura P. (2013). Moisture dependence of thermal expansion in cement-based materials at early ages. Cem. Concr. Res..

[43] Ulm F.J., Coussy O. (2001). What is a “massive” concrete structure at early ages? Some dimensional arguments. J. Eng. Mech..

[44] Wyrzykowski M., Lura P. (2013). Controlling the coefficient of thermal expansion of cementitious materials—A new application for superabsorbent polymers. Cem. Concr. Compos..

[45] Zahabizadeh B., Edalat-Behbahani A., Granja J., Gomes J.G., Faria R., Azenha M. (2019). A new test setup for measuring early age coefficient of thermal expansion of concrete. Cem. Concr. Compos..

[46] Loser R., Münch B., Lura P. (2010). A volumetric technique for measuring the coefficient of thermal expansion of hardening cement paste and mortar. Cem. Concr. Res..

[47] Kada H., Lachemi M., Petrov N., Bonneau O., Aïtcin P.C. (2002). Determination of the coefficient of thermal expansion of high performance concrete from initial setting. Mater. Struct..

[48] Bažant Z.P., Baweja S. (1995). Justification and refinements of model B3 for concrete creep and shrinkage 1. statistics and sensitivity. Mater. Struct..

[49] Barr B., Hoseinian S.B., Beygi M.A. (2003). Shrinkage of concrete stored in natural environments. Cem. Concr. Compos..

[50] Kockal N.U., Turker F. (2007). Effect of environmental conditions on the properties of concretes with different cement types. Constr. Build. Mater..

[51] Asamoto S., Ohtsuka A., Kuwahara Y., Miura C. (2011). Study on effects of solar radiation and rain on shrinkage, shrinkage cracking and creep of concrete. Cem. Concr. Res..

[52] Müller H.S., Pristl M., Bažant Z.P., Carol I. (1993). Creep and shrinkage of concrete at variable ambient conditions. Proceedings of the Fifth International RILEM Symposium on Creep and Shrinkage of Concrete (ConCreep 5).

